# Restricting social networking site use for one week produces varied effects on mood but does not increase explicit or implicit desires to use SNSs: Findings from an ecological momentary assessment study

**DOI:** 10.1371/journal.pone.0293467

**Published:** 2023-11-08

**Authors:** Michael Wadsley, Niklas Ihssen

**Affiliations:** Department of Psychology, Durham University, Durham, United Kingdom; Universita Cattolica del Sacro Cuore Sede di Roma, ITALY

## Abstract

Recent research on the addictive potential of modern technologies such as the internet, smartphones, or social networking sites (SNSs) has suggested that emotional and motivational changes associated with the sudden discontinuation of engagement with the technology mirror the aversive consequences seen when drug use is ceased abruptly. This has been observed even in moderate users and interpreted as a manifestation of withdrawal, an important marker of physical dependence in substance use disorders. On the other hand, a growing literature emphasises the positive effects of “digital detox” on mental health and well-being. Using a battery of affective and motivational measures, both explicit and implicit, the present study tracked the effects of a week of significantly reduced SNS use in moderate to heavy SNS users (N = 51). Our pre-registered analyses showed that the intervention elicited affective changes neither consistent with a general withdrawal syndrome (i.e., increased negative affect and cravings) nor with a general beneficial effect on well-being: While our data indicated some abstinence-related decreases of negative affect and boredom, they also showed a reduction of positive affect. These changes occurred regardless of problematic/addictive use behaviours. Importantly, restricting SNS use for one week had no effect on implicit measures of SNS use motivation (i.e., approach biases, time distortion and effort expenditure for SNS access) nor did it influence explicit cravings and SNS cue-reactivity. Together our findings suggest that restricting SNS use has nuanced and potentially offsetting effects on well-being. These could arise because use reduction may concurrently remove experiences that trigger negative emotions (e.g., upward social comparisons or Fear of Missing Out) but also those that elicit positive emotions (e.g., social approval). The hypothesised lack of a net effect on well-being would be consistent with our finding that voluntary reduction of SNS use does not mitigate or exacerbate SNS-related urges.

## Introduction

Excessive use of social networking sites (SNSs) is widely understood to have harmful consequences to our mental health and well-being [1, 2, but see 3], and numerous studies have shown that some SNS users display patterns of behaviour that resemble diagnostic criteria for traditional substance use disorders [4, 5]. Nonetheless, no formal diagnosis for ‘SNS addiction’ currently exists and many researchers dispute whether certain excessive behaviours should be conceptualised as addictions, fearing that this could lead to an over-pathologization of normal everyday activities [6, 7]. To illustrate this view, Satchell et al. [8] applied diagnostic criteria typically used to detect SNS addiction to offline social activities and found that 69% of individuals could be classified as addicted to spending time with their friends. While there is rightly no desire for ‘friend addiction’ to be recognised as a mental disorder, these findings may indicate that scales measuring ‘SNS addiction’ may not capture a clinically relevant pathology. Thus, the existence of ‘SNS addiction’ should be treated with a degree of scepticism when diagnoses are based solely on criteria adapted from scales used to diagnose substance use disorders without proper validation. More theory-driven, empirical research is needed to better understand whether certain SNS users do indeed display a similar symptomatology to that of individuals affected by substance use disorders.

Given the controversy surrounding the conceptualisation of a ‘SNS addiction’ previous research has used the term ‘problematic SNS use’ to refer to maladaptive use behaviours without assuming the presence of an addiction. Therefore, we also adopt the term ‘problematic SNS use’ in this report to refer to SNS behaviours that are frequently used as diagnostic markers of ‘SNS addiction’ in assessment instruments (e.g., use that causes issues at work/school etc.). Typically, the diagnosis of ‘SNS addiction’ relies on adjusted versions of the DMS-5’s proposed criteria for internet gaming disorder [9]. These include *preoccupation* (i.e., spending a lot of time thinking about SNS use or planning the next use), *tolerance* (i.e., a need to spend increasing amounts of time using SNSs), *withdrawal* (i.e., experiencing negative psychological or physical effects when attempting to cut down or stop SNS use), *loss of control* (i.e., unsuccessful attempts to cut down or stop SNS use), *displacement* (i.e., losing interest in previous hobbies/activities because of SNS use), *problems* (i.e., continued SNS use despite the knowledge of negative consequences), *deception* (i.e., lying to others about using SNSs), *mood modification* (i.e., using SNSs to escape from dysphoric mood), and *conflict* (i.e., risking or losing a significant relationship, job, education or career opportunity because of SNS use). However, many of these criteria have been criticized for lacking diagnostic validity and pathologizing normal use motives [10–12]. In particular, the relevance of withdrawal for behavioural addictions is poorly understood [13]. Because behavioural addictions do not involve the ingestion of a psychoactive substance, the experience of severe physical withdrawal symptoms is unlikely to occur [14]. Instead, withdrawal in behavioural addictions is thought to manifest as negative emotional states (e.g., feeling sad, anxious, angry) and to result in an increased motivation for the activity (e.g., increased subjective cravings and implicit responses to addiction-related cues). Importantly, to be considered genuine withdrawal symptoms these changes in emotional/motivational states should occur for at least several hours/days after the behaviour is ceased so as to avoid conflating withdrawal with normal responses to ending a pleasurable activity [12]. In addition to withdrawal, attempts to abstain from an addictive substance can also be expected to result in relapses and rebound effects. That is, individuals with an addiction experience an inability to maintain drug abstinence (i.e., relapse) and increased drug consumption compared to baseline after a temporary period of full or partial abstinence (i.e., rebound). As such, if problematic SNS use warrants recognition as an addictive disorder then similar experiences can be expected to occur in more problematic users who undergo a period of SNS abstinence or use reduction.

Indeed, a number of studies have found negative, potentially withdrawal-like, outcomes from SNS abstinence. One key feature of withdrawal is an increase in cravings when the drug/behaviour is unavailable [15–17]. In line with this, when abstaining from SNSs for one week, Stieger and Lewetz [18] found that participants experienced increases in cravings, boredom and social pressure to use SNSs, as well as (nonsignificant) reductions in positive and negative affect. In addition to these withdrawal-like effects the researchers also report that around 60% of participants ‘relapsed’ at least once during the intervention, suggesting that the majority of SNS users experience difficulties controlling their use behaviours. Other potential withdrawal effects resulting from a week of SNS abstinence have been shown to include a decline in life satisfaction and increases in negative affect and loneliness [19]. During a 48 hour period of Facebook abstinence Sheldon et al. [20] found that participants reported feeling less connected, and the experience of greater disconnection during abstinence predicted more excessive Facebook use in the future. Additionally, Hanley et al. [21] showed that the experience of withdrawal effects may depend on how individuals use SNSs. They found that active, but not passive SNS users reported reduced positive affect after abstaining from Facebook and Instagram for one week. According to the authors, active users may be more likely to experience benefits from their SNS use, but reduced positive affect resulting from SNS abstinence could also reflect a withdrawal-like symptom in these users. However, all of these studies employed samples of regular (nonproblematic) SNS users and did not correlate affective measures with symptoms of problematic use. Withdrawal effects can also be inferred from assessments of time distortion. Turel and Cavagnaro [22] showed that when participants completed a task requiring them to reflect on their SNS use, those who had abstained from SNSs for a week displayed a significantly larger upwards time distortion bias (i.e., believing more time had passed than actually had) compared to those who had not abstained. Furthermore, this effect was more pronounced in individuals with more addiction-like symptoms. The authors argued that more addicted individuals had experienced more negative emotions (e.g., increased stress and cravings) when deprived from using SNSs, making it seem as though the task had taken longer than it actually did [23].

Conversely, a period of SNS abstinence can also be predicted to alleviate the harmful psychological effects associated with SNS use, producing positive outcomes. In one of the most comprehensive studies to investigate the effects of SNS abstinence it was found that deactivating Facebook for a period of four weeks increased subjective well-being and reduced post-experiment Facebook use [24]. Furthermore, another large-scale study showed that after a week of Facebook abstinence participants reported increases in life satisfaction and positive affect [25]. Fioravanti et al. [26] showed that quitting Instagram for a week increased life satisfaction and positive affect, but only for women with a tendency towards making social comparisons. The positive effects associated with a week of SNS abstinence have also been shown to include a reduction in the ‘fear of missing out’ (FoMO) and increased social connectedness [27]. One study has even shown that five days of SNS abstinence may have positive physiological effects [28]. While Facebook abstinence did not affect perceived stress in this study, participants who abstained did show reduced cortisol levels (the body’s primary stress hormone). However, despite this reduction in physiological stress, Facebook abstinence also resulted in lower self-ratings of life satisfaction. In the only study to find positive outcomes from SNS abstinence in more problematic SNS users, Turel et al. [29] found that both problematic and regular users experienced a reduction in perceived stress during abstinence, with this effect being more pronounced in problematic users. Such a finding therefore directly contradicts the concept of a SNS withdrawal syndrome as increased stress would be predicted in individuals with more addiction-like symptoms. Other studies have shown that limiting SNS use, rather than quitting completely, can result in similar beneficial effects in regular SNS users. Brailovskaia et al. [30] found that Facebook users who restricted their use to 20 minutes per day for two weeks reported increased well-being and reduced depressive symptoms. Similarly, SNS users who limited their use to 30 minutes per day over a period of three weeks experienced reduced loneliness and depression [31]. Consistent with the positive effects of use reduction, Graham et al. [32] found that limiting daily SNS use to 10 minutes per app for one week improved well-being and sleep quality.

Nonetheless, other research has failed to observe any effects of SNS abstinence or use reduction on mood and well-being. In one such study participants were randomly assigned to one of five experimental conditions, consisting of varying abstinence durations (1–4 weeks) and a control group continuing with their regular use [33]. Surprisingly the researchers found that SNS abstinence, even after four weeks, had no effect on daily measures of subjective well-being, loneliness and quality of day. Collis and Eggers [34] also report no effects of restricting SNS use. When participants limited their SNS use to 10 minutes per day for a period of nine weeks, no effect on their well-being or academic success was observed. Similarly, van Wezel et al. [35] found no effects on well-being or behavioural measures of attention when participants reduced their SNS screentime by 50%. Other research using a shorter abstinence manipulation has shown that preventing SNS use for one day has no effect on well-being, although some individuals showed reduced social relatedness and satisfaction with one’s day [36]. Schwarz et al. [37] also report that while participants who abstained from Instagram for one week showed improvements in general mental state and self-esteem as well as a reduction in depressive symptoms compared to baseline, these improvements did not significantly differ from those observed in a control group who did not restrict their Instagram use. However, none of the studies that report null effects from SNS abstinence employed a measure of problematic SNS use.

As outlined above, the available literature appears inconsistent regarding the potential beneficial and detrimental effects of SNS use cessation or reduction. This is corroborated by a recent systematic review of digital detox interventions, showing that previous research has reported both positive and negative effects of abstinence on measures of mood and well-being, with other studies finding no effects at all [38]. Variability in the reported effects may partly be accounted for by differences between studies in terms of abstinence requirements (complete abstinence vs restricted use) and duration, the focus on different SNS platforms and other factors. Furthermore, very few studies have investigated the effects of SNS abstinence/use reduction contingent on problematic use symptoms. Since the experience of withdrawal might only be expected to occur in more problematic users, it is important for research to take the presence of addiction-like symptoms into account when assessing the concept of a SNS withdrawal syndrome.

In the present study we tracked self-reported affect (i.e., positive, and negative emotions) and motivation (i.e., SNS cravings) on a day-by-day basis using ecological momentary assessments (EMAs) across a 7-day intervention which asked participants to abstain from SNS use. We also included separate assessments of boredom and loneliness since previous research has indicated that such states might be associated with SNS abstinence or use reduction [18, 19]. To allow for the assessment of potential rebound effects and compensatory behaviours the present study also employed baseline (3-days) and post-intervention (4-days) assessments. A recent systematic review of abstinence effects across different behavioural addictions has indicated that rebound effects and compensatory behaviours have not been adequately assessed in previous research [39]. Such assessments are important as short-term SNS abstinence interventions may not be advisable if individuals substitute their SNS use for equally harmful activities or if the harmful consequences of their SNS use are exacerbated when normal use is resumed. For example Collis and Eggers [34] found that when students had to limit their SNS use they spent more time using instant messaging apps and did not reduce their overall digital screen time. In fact, the participants in the abstinence condition overcompensated for their lack of SNS use and spent significantly more time using digital devices than those in a control group. There is also some evidence that reducing online activity increases TV watching [24] but may also encourage beneficial behaviours such as increasing exercise and reducing smoking [30].

Most previous research using abstinence/use reduction interventions also suffers from a reliance on self-report assessments of emotional and motivational states. In the present study, we therefore included a number of lab-based experimental measures to gauge the effects of the intervention period on implicit use motivation. In substance use disorders, such measures (e.g., unconscious changes in drug wanting) have been shown to be associated with the experience of withdrawal, with increased attentional biases to drug-related stimuli reflecting the increased incentive salience attributed to these cues during a state of drug deprivation [40, 41]. In behavioural addictions implicit measures of reward processes have also been used to demonstrate modifications of attentional bias to gambling cues in abstinent pathological gamblers [42]. Yet, in this study abstinent pathological gamblers displayed an avoidance bias to gambling cues. This may reflect that pathological gamblers would need to effortfully inhibit gambling cravings/urges during abstinence, which might facilitate strategies to intentionally avoid gambling cues. More recently studies have begun to establish the relevance of implicit processes (e.g., implicit attitudes, attentional biases, approach-avoidance tendencies) for problematic SNS use [43–48]. Implicit use motivation also appears to characterise SNS use more generally, and we have recently demonstrated the existence of increased implicit approach tendencies towards SNS stimuli in a large sample of young adults [49]. However, to our knowledge only two studies have employed implicit measures to investigate effects of SNS abstinence/use reduction (i.e., time distortion [22] and sustained attention [35]). In two lab sessions immediately before and after the abstinence intervention, the present study assessed implicit SNS wanting using an adapted Visual Approach/Avoidance by the Self Task [VAAST; 50], an assessment of time distortion, and an adapted Effort Expenditure for Reward Task [EEfRT; 51]. Furthermore, using a visual cue reactivity task the lab sessions also assessed changes in explicit motivational responses to SNS app icons that might be modified by a period of abstinence.

For the EMA measures we predicted that abstinence would reduce positive affect and increase negative affect from baseline (t1) to the intervention period (t2) and that these effects would be correlated with more problematic SNS use (Hypothesis 1a). We also predicted that abstinence would increase self-reported experiences of boredom, loneliness and cravings to use SNSs from t1 to t2 and that these effects would be correlated with more problematic SNS use (Hypothesis 1b).

For the experimental measures obtained in the two lab sessions we expected that SNS deprivation during the intervention period would result in increased implicit and explicit motivation for SNS use. Specifically, we predicted that visual cue reactivity to SNSs would be stronger at session 2 (last day of intervention period) compared to session 1 (first day of intervention; Hypothesis 2a). We also hypothesised that larger cue reactivity scores after abstinence would be correlated with more problematic SNS use (Hypothesis 2b). Further, we predicted that the approach bias to SNS stimuli in the social media VAAST would be greater at session 2 compared to session 1 (Hypothesis 3a) and that a stronger approach bias to SNS stimuli after abstinence would be correlated with more problematic use (Hypothesis 3b). For the time distortion task, we predicted a stronger time distortion effect at session 2 compared to session 1 (Hypothesis 4a) and that this effect would be correlated with more problematic SNS use (Hypothesis 4b). Finally, we predicted that participants would exert more effort in the adapted EEfRT at session 2 compared to session 1 (Hypothesis 5a) and that these effects would also be correlated with more problematic SNS use (Hypothesis 5b). The method and hypotheses for this study were preregistered on the Open Science Framework (https://osf.io/pe7aw).

## Method

### Participants

Fifty-five young adults between the ages 18–25 were recruited from the student population at Durham University and took part in the study between January-June 2022. Three participants withdrew from the study and data from one participant was excluded from the analysis as they did not meet the inclusion criteria (iPhone user). The final sample of 51 participants (M_age_ = 19.92, SD = 1.16, 16 males, 35 females) all reported using at least one SNS daily, using an iPhone with the Screen Time app enabled, and being willing to abstain from using SNSs for one week. Additionally, 16 participants were excluded from analysis of the mood data due to inadequate survey response rates (see results section, final N = 35 for analysis of mood changes), and three participants were excluded from analysis of the VAAST data due to insufficient response accuracy (< 60% of trials correct; final N = 48). To ensure the sample included participants with varying levels of problematic use, separate adverts were used to target more problematic users (those who score 4 or more on the Social Media Disorder Scale; SMDS; [9]) and less problematic users (those who score less than 4 on the SMDS). Participants received course credits or £30 Amazon vouchers for their participation. The study was approved by the Ethics Sub-Committee in the Department of Psychology at Durham University on 8^th^ March 2021 (PSYCH-2021-01-25T15_08_42-hxck16) and all participants provided written informed consent.

### Procedure

Ecological momentary assessments (EMAs) of mood and urges to use SNSs were collected across a period of 15 days. The experimental design comprised a 3-day baseline phase (normal SNS use), 6-day intervention phase (SNS abstinence) and 4-day post-intervention phase (normal SNS use). EMA data from the start and end dates of the intervention period (i.e., 4^th^ and 11^th^ days) were not included in the pre-registered analysis, since these days consisted of both abstinence and normal use periods. Upon signing up to the study participants were required to download the SEMA^3^ app on their mobile device (https://sema3.com/), which was used to administer the EMAs and delivered notifications 3 times a day (at random times between 10 AM and 9 PM). Participants had 30 mins to complete an assessment after receiving a notification. To avoid the occurrence of successive notifications within a short period, one EMA was delivered during three separate time windows (10:00–13:00, 14:00–17:00, 18:00–21:00). In addition to the EMAs, participants also received a notification at 21:30 each day to complete an end‐of‐day questionnaire and had a 2-hour period in which to complete it.

On the 4th day of the study participants were invited into the psychology department to complete the first lab experiment. During this session participants completed computer-based experiments including measures of approach-avoidance tendencies in relation to SNS stimuli, time perception and effort expenditure for SNS access, as well as a SNS cue reactivity task and questionnaire measures. The effort task was programmed using PsychoPy [52] and all other lab measures were programmed using PsyToolkit [53, 54]. At the end of the session participants were instructed to abstain from using all SNSs (e.g., Instagram, Facebook, Twitter; but not including instant messaging or voice/video calling apps e.g., WhatsApp or Messenger) for the next 7 days. Participants were advised to turn off SNS notifications and to prevent/limit access to SNS apps using the iPhone Screen Time app. However, participants were not instructed to delete these apps to avoid participants using other devices to access their SNSs. By leaving SNSs functional, associated screen time data was not deleted, and we could thus track any “relapse” periods more accurately.

Seven days after the first session (11th day of the study) participants completed the second lab session. Both sessions took place at the same time of day and in the same environment. The measures in the second session were identical to those in the first session except that the questionnaire measures were not repeated. At the end of the session participants also provided ratings of familiarity, arousal, and valence for the SNS and control stimuli used in the VAAST. After the second session was completed, participants were told that they could resume using SNSs as normal. The researcher had access to information that could identify individual participants during the data collection period, but all data were made anonymous at the end of the study.

### Materials

#### Ecological momentary assessments

Daily questionnaires comprised five dependent measures (nine items in total), assessing similar affective and motivational variables as comparable abstinence studies [18]: (1) Boredom (“How bored do you feel right now?”), (2) loneliness (“How lonely do you feel right now?”) and (3) SNS cravings (“How much do you want to use social media right now?”) were assessed using single item measures. Additionally, three questions assessing (4) positive affect and three questions assessing (5) negative affect were taken from the International Positive and Negative Affect Schedule-Short Form [I-PANAS-SF; 55]. These questions asked participants to report their current happiness, cheerfulness and liveliness (positive affect) as well as sadness, miserableness and madness (negative affect). Responses to each question were made using a 0–100 sliding scale (0 = not at all, 100 = very bored/lonely/happy etc.) and question order was randomised for each questionnaire.

#### End-of-day questionnaire

An end-of-day questionnaire was used to obtain daily measures of SNS screen time, total iPhone screen time and reports of any behaviours that participants had used to compensate for the non-use of SNSs. When reporting their screen times, participants were always instructed to report their usage from the previous day using the Screen Time app on their phone in order to ensure that each measure represented a whole 24-hour period. Thus, for measures of screen time, the baseline phase was 4 days, and the post-intervention phase was 3 days. Participants were first asked to use the Screen Time app to report the number of minutes spent on SNSs during the previous day. When calculating their SNS screen time participants were asked to subtract any time spent using instant messaging apps (e.g., WhatsApp) but to include any time spent on SNSs not listed under the ‘social’ category in the Screen Time app (e.g., YouTube, categorised as ‘entertainment’). They were then asked to estimate any additional time spent using SNSs on other devices during the previous day. These two measures were summed to calculate daily SNS usage. An additional question asked participants to use the Screen Time app to report their total iPhone screen time on the previous day. A final question asked participants to report potential compensatory behaviours by asking “Have you done any of the following activities today more than you would do usually?”. Participants responded to six categories of activities and rated the extent of their engagement for activities they reported doing more than usual using a 1–7 sliding scale (1 = slightly more than usual, 7 = much more than usual). The assessed compensatory behaviours consisted of 1) Watching TV or video streaming sites (e.g., Netflix); 2) Eating junk food; 3) Playing video games; 4) Drinking alcohol; 5) Gambling; 6) Online shopping. In cases where participants did not complete an end-of-day questionnaire the researcher followed up with them to obtain the screen times for the missed days. However, participants were not asked to provide retrospective estimates of compensatory behaviours.

#### Visual cue reactivity

Similar to our previous research [49, 56], participants were asked to rate the extent to which images of 6 SNS icons (Facebook, Instagram, Snapchat, Twitter, TikTok and YouTube) and 6 iPhone app control icons (Settings, Maps, App Store, Photos, Weather, Books) made them want to check the corresponding application. Participants were asked to respond to each statement (e.g., “this icon makes me want to check the Facebook app?”) using a 7-item Likert scale (strongly disagree–strongly agree). Cue reactivity scores were calculated at pre- and post-intervention by subtracting the average response to control icons from the average response to the SNS icons. During the second session participants were also asked to provide ratings of familiarity (“I am familiar with this icon”), valence (“this icon is visually appealing”), and arousal (“this icon is exciting”) for each icon. Participants were instructed to respond to each statement with regard to the image itself rather than how much they liked a specific app.

#### Approach/Avoidance task

Participants completed an adapted Visual Approach/Avoidance by the Self Task [VAAST; 50] with SNS and control stimuli, previously described in Wadsley and Ihssen [49]. In this task participants were required to move towards or away from SNS or control icons using the computer keyboard. Approach vs avoidance movements were reflected by increases/decreases in the size of the visual stimuli. Participants were instructed to approach SNS icons and avoid control icons in the first block, and vice versa for the second block, while others complete the same blocks in the reversed order (counterbalanced across participants). The block order for each participant was the same across the two testing sessions. The SNS stimuli consisted of Facebook, Instagram, Twitter, Snapchat, TikTok and YouTube app icons, whereas the control stimuli consisted of Maps, Weather, App Store, Books, Photos and Settings iPhone app icons. A training phase with feedback occurred before the experimental trials in each block. The training phase consisted of 12 trials where every stimulus was presented once in a random order (i.e., 6 SNS and 6 control stimuli). During each trial an image of a hand holding a smartphone is displayed on a grey background. Participants pressed the ’H’ key to initiate the trial. A fixation cross was then shown in the middle of the screen for a random duration between 800-2000ms at intervals of 100ms, followed by the stimulus presentation which remained on screen until a response was made. Participants pressed the ’Y’ key to approach or the ’N’ key to avoid and were instructed to only use the index finger of their dominant hand when making responses. An inter-stimulus-interval of 500ms occurred after each response. In the experimental phase a total of 60 trials occurred in a random order for each block (participants completed a total of 144 trials, including 2 × 60 experimental trials and 2 × 12 training trials).

Approach/avoidance bias scores were calculated for each participant at pre- and post-intervention for SNS (RTSNS_Avoid–RTSNS_Approach) and control stimuli (RTControl_Avoid–RTControl_Approach), whereby positive scores indicate an approach bias, and negative scores indicate an avoidance bias. Overall AAT scores were calculated at pre- and post-intervention using the formula [(RTSNS_Avoid–RTSNS_Approach)–(RTControl_Avoid–RTControl_Approach)], with positive scores indicating a stronger approach bias to SNS stimuli relative to control stimuli.

#### Time distortion

Immediately after completing the VAAST participants were asked to estimate to the nearest minute, how long it took to complete the task. The actual time taken to complete the VAAST was automatically recorded to the nearest minute. Time distortion was calculated at pre- and post-intervention by subtracting the actual time from the participant’s estimated time taken to complete the task, with 0 representing no bias, positive values representing an overestimation and negative values representing an underestimation.

#### Effort Expenditure for Reward Task

Participants completed a modified version of the Effort Expenditure for Reward Task [EEfRT; 51]. Here participants worked to gain brief exposure to their SNS accounts. On each trial participants chose between completing an easy task (repeatedly pressing the spacebar with the index finger of their dominant hand) or hard task (repeatedly pressing the spacebar with the little finger of their non-dominant hand). Participants had the opportunity to practice both the hard and easy task before completing 12 experimental trials. To win the easy task participants were required to press the spacebar more than 35 times in 7 seconds whereas the hard task required more than 70 presses in 15 seconds. However, participants were not told the number of presses required to win each task. A win on the easy task rewarded the participant with 10 seconds on a SNS, whereas a win on the hard task rewarded 30 seconds on a SNS. A fail on either task resulted in 0 seconds to spend on SNSs. Unlike the original EEfRT [51], in this simplified adaptation there was no variation in reward value or probability of winning. In order to make the possibility of gaining access to their SNSs more salient, before beginning the task participants were told to place their phone face down on the desk next to them and they were informed that they would not be allowed to touch their phone again until after the task. After completing the 12 experimental trials there was a 6-minute period during which participants were able to use the time they had accumulated in the task to spend on a SNS of their choice. However, participants were informed at the beginning of the task that they would have to wait in silence for the duration of the 6-minutes that they had not won time to spend on SNSs. For example, a participant who selected and won the easy task on each of the 12 trials would have won 2 minutes to spend on a SNS (12 × 10 secs). Therefore, they would first have to wait 4 minutes before being allowed to access a SNS on their phone for the remaining 2 minutes. The waiting period served as a ‘punishment’ for not choosing the hard task or failing to exert sufficient effort and was designed to incentivise those participants with higher SNS cravings to select and exert more effort on the hard task. Thus, task choice and number of spacebar presses provided an implicit measure of SNS ‘wanting’.

#### Lab-based questionnaire measures

We obtained a measure of problematic SNS use using the SMDS [9], which is a 9-item scale based on the 9 suggested DSM-5 criteria for Internet Gaming Disorder. Participants were also asked to self-report the frequency that they check their SNS accounts (7-item Likert scale, less than daily–every 15 mins). Measures relating to the number of different SNS platforms used, the expected number of ‘likes’ on a typical SNS post, and general sensitivity to reward [assessed using 10 questions taken from the SPSRQ-20; 57] were also obtained but were not further analysed.

## Results

### Descriptive statistics

**Fig 1:**
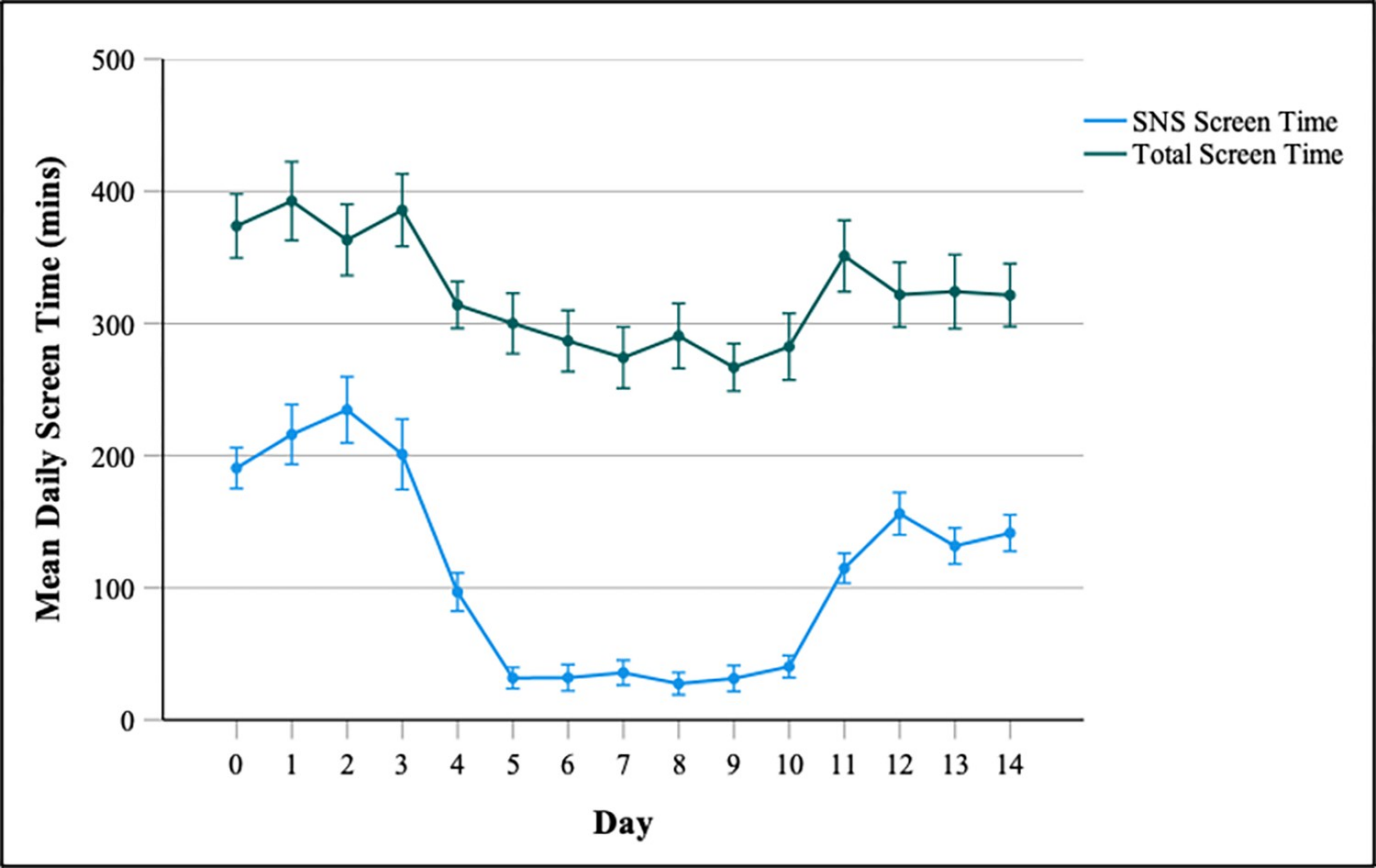
Day-by-day changes in screen times (mins) for SNS use and iPhone use. The abstinence intervention began midway through day 4 and ended midway through day 11. Error bars represent +/- 1 SE mean.

SMDS scores in our sample ranged from 1–6 (M = 3.57, SD = 1.59) with 17 participants (33.3%) scoring 5 or 6 (thus meeting the proposed cut-off score for ‘disordered SNS user’; [9]). Self-reported checking frequency ranged from 2 (every day)-7 (every 15 minutes) (Mdn = 6 [every 30 minutes], IQR = 1). Mean daily SNS and smartphone screentime for each phase of the experiment are displayed in Table 1, and day-by-day changes in screen time are displayed in Fig 1.

**Table 1:** Mean daily screen times (mins) at each phase of the experiment for SNS use and iPhone use.

	Baseline (t1)		Intervention (t2)		Post-intervention (t3)	
	Mean (SD)	Range	Mean (SD)	Range	Mean (SD)	Range
SNS screen time	208 (115)	30–570	35 (53)	0–245	146 (82)	12–300
Total smartphone screen time	378 (153)	95–791	283 (139)	62–891	324 (156)	64–769

All but two participants reported reduced SNS use during the intervention period compared to baseline. The two participants who reported higher SNS use during the intervention were not removed since their performance would still reflect an effect of attempting to abstain from SNSs. We ran two separate repeated measures ANOVAs for SNS screen time and total iPhone screen time, with experiment phase as the within-subjects factor (baseline vs. intervention vs. post-intervention). The ANOVA for SNS screen time revealed a significant effect of experiment phase [*F*(2, 100) = 68.81, *p* < .001, η_p_^2^ = 0.579]. Follow up paired t-tests revealed that the average daily SNS screen time during the intervention period was significantly lower than at baseline [*t*(50) = 10.93, *p* < .001]. During the post-intervention phase SNS screen time became significantly higher than during the intervention phase [*t*(50) = -9.00, *p* < .001] but was still significantly lower than during baseline [*t*(50) = 3.78, *p* < .001]. Similarly, the ANOVA for total iPhone screen time also revealed a significant effect of experiment phase [*F*(2, 100) = 18.20, *p* < .001, η_p_^2^ = 0.267]. Follow up paired t-tests revealed a similar pattern with all differences between the three time points being significant (*p* < 0.01).

We also examined the number of participants who ‘relapsed’ during the intervention period. While most participants were able to substantially reduce their SNS usage during the intervention, only 7 participants (13.7%) managed to successfully abstain for the full week. Nonetheless, despite low compliance with the abstinence instruction, during the intervention phase participants did reduce their SNS use from baseline by an average of 83.4%.

### Ecological momentary assessments

Only participants who responded to at least 50% of the surveys at each phase of the experiment were included in the analysis, resulting in the exclusion of 16 participants. Of the remaining 35 participants, the mean response rate was 78.1% (SD = 11.71) and response rates did not significantly differ between the three experiment phases [*F*(2, 68) = 1.19, *p* = .311, η_p_^2^ = 0.034]. Data from the 4^th^ and 11^th^ days were discarded owing to the fact that participants began their week of SNS abstinence at different times of the day.

We ran separate repeated measures ANOVAs for each measure of mood, with experiment phase as the within-subjects factor (baseline vs. intervention vs. post-intervention). Where Mauchly’s test indicated that the assumption of sphericity had been violated we used the Greenhouse-Geisser corrected degrees of freedom. The ANOVA for positive affect revealed a significant effect of experiment phase [*F*(1.61, 54.55) = 8.92, *p* = .001, η_p_^2^ = 0.208]. In contrast, no significant effect of experiment phase was observed for negative affect [*F*(1.61, 54.73) = 2.38, *p* = .112, η_p_^2^ = 0.065], boredom [*F*(1.55, 52.75) = 3.24, *p* = .059, η_p_^2^ = 0.087], loneliness [*F*(2, 68) = 1.37, *p* = .261, η_p_^2^ = 0.039], or cravings [*F*(2, 68) = 1.09, *p* = .342, η_p_^2^ = 0.031].

**Fig 2:**
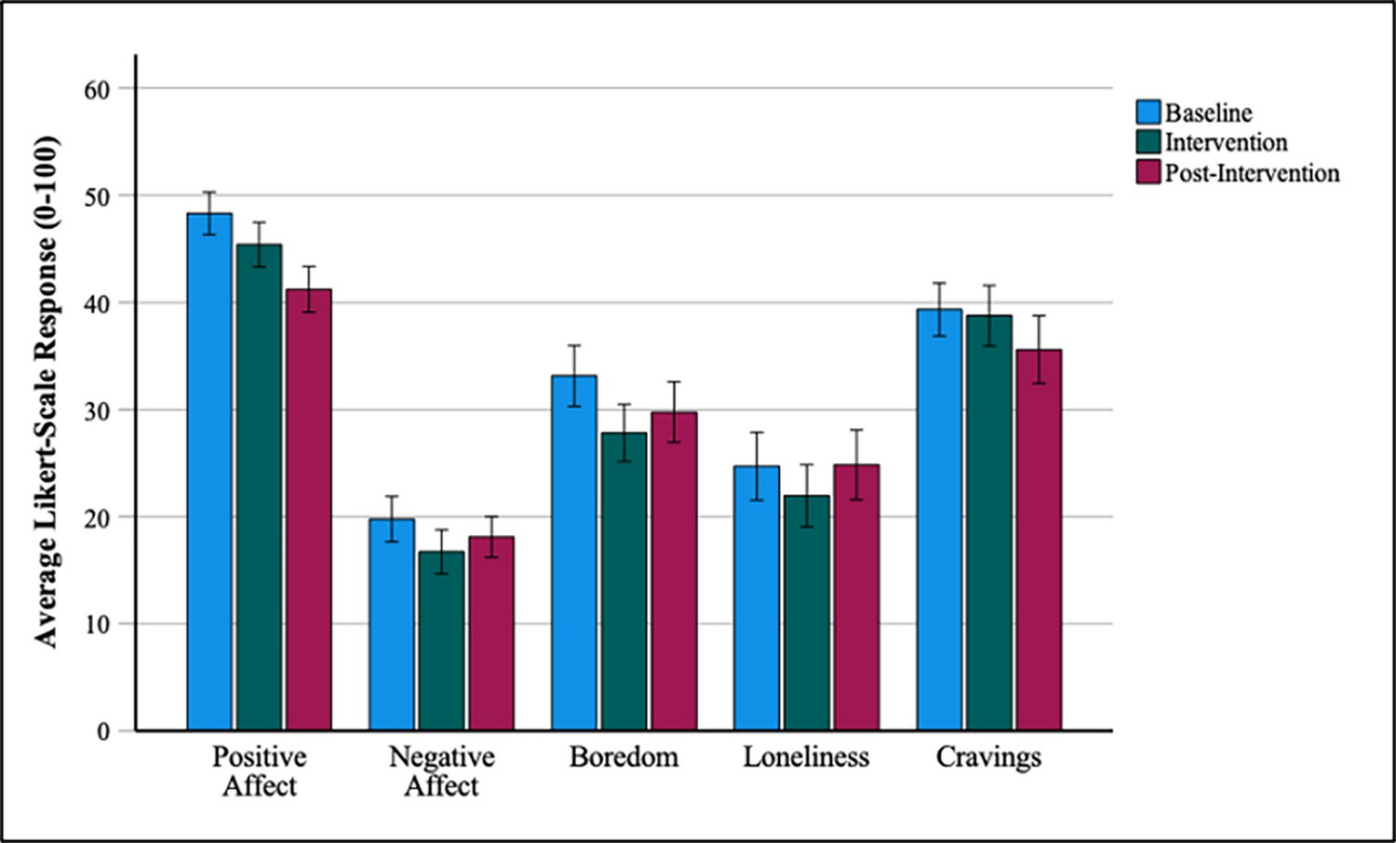
Mean self-report ratings of mood across the three experiment phases. Error bars represent +/- 1 SE mean.

Follow up paired t-tests were conducted to explore the effect of experiment phase on positive affect. This revealed a significant decrease in positive affect from intervention (M = 45.38, SD = 12.31) to post-intervention (M = 41.22, SD = 12.63) [*t(*34) = 3.42, *p* = .002, d = 0.58]. Positive affect at baseline (M = 48.32, SD = 11.75) was also significantly higher than at post-intervention [*t(*34) = 3.62, *p* = .001, d = 0.61]. While positive affect was reduced from baseline to intervention, this difference did not reach significance [*t(*34) = 1.64, *p* = .111, d = 0.28]. Exploratory paired t-tests also revealed marginally significant reductions in negative affect [*t(*34) = 2.06, *p* = .047, d = 0.35] and boredom [*t(*34) = 2.24, *p* = .032, d = 0.38] from baseline to intervention. All other comparisons were nonsignificant. Mean scores for each measure of mood are displayed in Fig 2.

For each mood measure ‘withdrawal scores’ were calculated by subtracting baseline ratings from ratings at the intervention phase. Similarly, ‘rebound scores’ were calculated by subtracting baseline ratings from ratings at the post-intervention phase. Withdrawal and rebound effects were correlated with SMDS scores, self-reported checking frequency and baseline SNS screen time. Negative affect rebound effects were weakly correlated with SMDS scores (r = -.34, *p* = .044), however the correlation did not survive a Bonferroni correction for multiple tests (adjusted α = .017). No other significant correlations were found.

### Visual cue reactivity

A paired t-test revealed no significant difference in cue reactivity scores between pre- and post-intervention [*t(*50) = -0.24, *p* = .811, d = 0.03]. The difference between pre- and post-intervention cue reactivity scores was also correlated with SMDS scores, self-reported checking frequency and baseline SNS screen time. However, no significant correlations were found.

### Approach/avoidance task

Only participants with an accuracy rate of 60% or more on both trial types (approach SNS condition and approach controls condition) were included in the analysis (see preregistration), resulting in the exclusion of three participants. The overall accuracy rate in the VAAST was high (M = 96.89%, SD = 3.13) and accuracy did not significantly differ between the pre- (M = 96.61%, SD = 4.15) and post-intervention sessions (M = 97.17%, SD = 3.14) [*t(*47) = -1.00, *p* = .323, d = 0.14]. Only RTs for correct responses were included in the analysis. Correct responses with RTs less than 200 ms or greater than 3000 ms were also removed as outliers, resulting in the exclusion of a further 0.41% of the trials.

**Fig 3:**
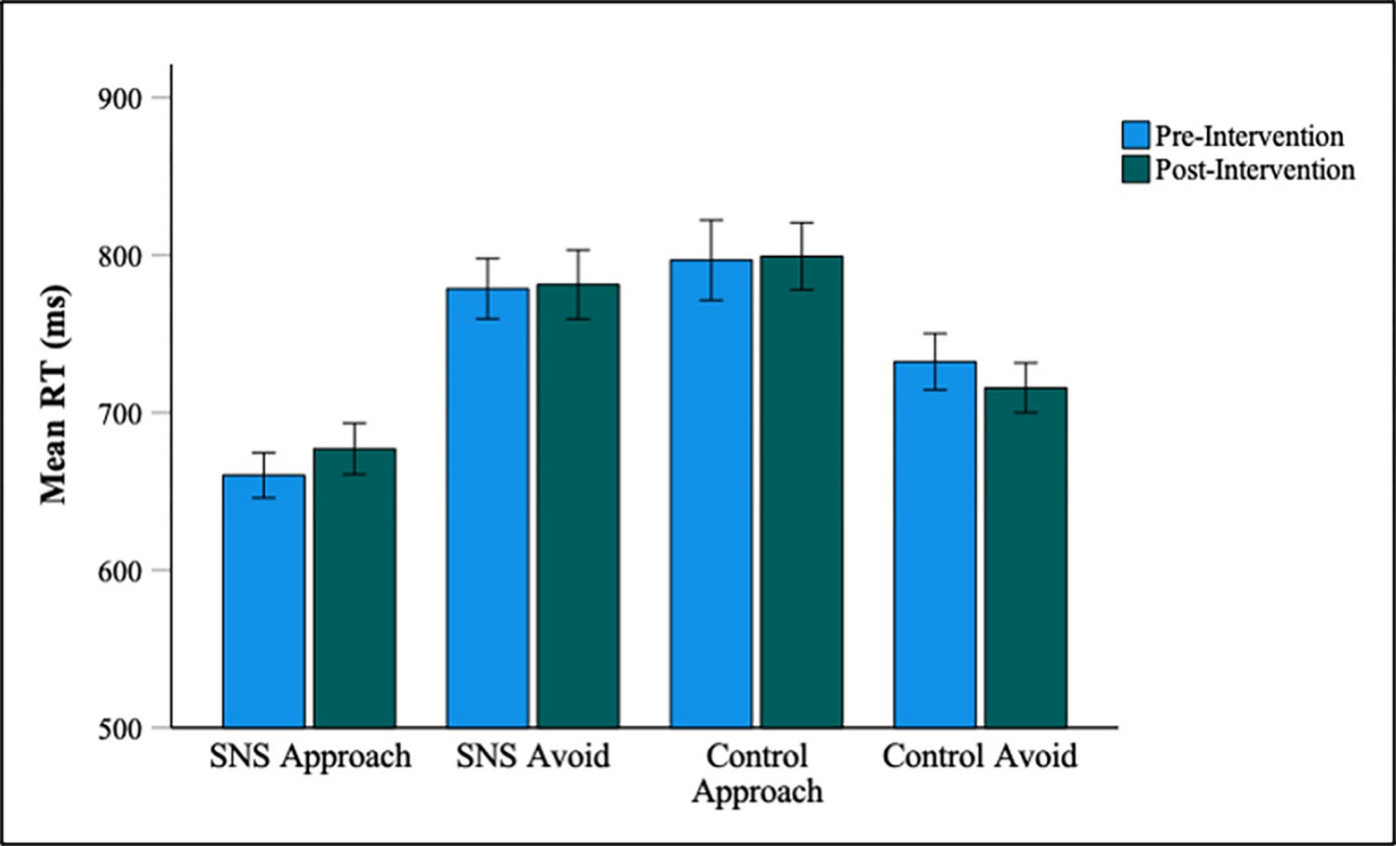
Mean VAAST RTs for each condition at pre- and post-intervention. Error bars represent +/- 1 SE mean.

To investigate the effect of abstinence on approach/avoidance biases, bias scores were analysed using a 2 (abstinence: pre vs. post) × 2 (category: SNS vs. control) repeated measures ANOVA. The ANOVA revealed a significant main effect of category, [*F*(1, 47) = 50.68, *p* < .001, η_p_^2^ = 0.519], whereby participants displayed an approach bias to SNS stimuli (M = 111.41, SD = 119.63) and an avoidance bias to control stimuli (M = -73.97, SD = 127.13). However, the main effect of abstinence was nonsignificant [*F*(1, 47) = 2.44, *p* = .125, η_p_^2^ = 0.049], as was the interaction effect [*F*(1, 47) = 0.02, *p* = .888, η_p_^2^ < 0.001]. Mean RTs for each condition are displayed in Fig 3.

The difference in overall AAT scores between the two sessions was also calculated (session 2 –session 1) and these scores were correlated with SMDS scores, self-reported checking frequency and baseline SNS screen time. Self-reported checking frequency (analysed using nonparametric Spearman’s Rho) was weakly correlated with overall AAT difference scores (r_s_ = .30, *p* = .041), however the correlation did not survive a Bonferroni correction for multiple tests (adjusted α = .017). No other significant correlations with overall AAT difference scores were found.

### Time distortion

The time taken to complete the VAAST ranged from 9–14 minutes, whereas participants estimated time taken to complete the VAAST ranged from 4–22 minutes (see Table 2). Participants became faster at completing the VAAST on their second attempt (post-intervention) [*t(*50) = 9.70, *p* < .001, d = 1.36] and correspondingly there was a marginally nonsignificant reduction in their time estimates [*t(*50) = 1.99, *p* = .052, d = 0.28]. Time distortion was calculated at pre- and post-intervention by subtracting the actual time from the participant’s estimated time taken to complete the task. While we did observe a larger upwards time distortion bias at post-intervention, the difference was nonsignificant [*t(*50) = 0.70, *p* = .488, d = 0.10]. The difference between pre- and post-intervention time distortion were also correlated with SMDS scores, self-reported checking frequency and baseline SNS screen time. However, no significant correlations were found.

**Table 2:** Descriptive stats for estimated VAAST time, actual VAAST time and time distortion measured in minutes.

Measure	Minimum	Maximum	Mean	SD
*Pre-intervention*				
Estimated time	4	22	11.43	4.02
Actual time	9	14	11.22	1.25
Time distortion	-8	11	0.22	3.90
*Post-intervention*				
Estimated time	5	18	10.43	3.42
Actual time	9	12	9.86	0.83
Time distortion	-4	7	0.57	3.30

### EEfRT

During the easy task participants pressed the spacebar an average of 41.08 times (SD = 5.47), whereas during the hard task the mean number of spacebar presses was 77.20 (SD = 9.47). The number of times participants chose to complete the hard task served as a measure of willingness to exert effort and the total number of keypresses served as a measure of actual effort expenditure. Contrary to what was predicted, participants selected the hard task on fewer occasions during the second session, although this difference was not significant [*t(*50) = 1.10, *p* = .278, d = 0.15]. Similarly, participants also exerted less effort in the task during the second session, but this difference was also not significant [*t(*50) = 1.67, *p* = .102, d = 0.23]. The difference between pre- and post-intervention (t2 –t1) hard task choices and total keypresses were also correlated with SMDS scores, self-reported checking frequency and baseline SNS screen time. Self-reported checking frequency (analysed using nonparametric Spearman’s Rho) was negatively correlated with the difference in total keypresses (r_s_ = -.30, *p* = .031), indicating that individuals who report checking SNSs more frequently exerted less effort on the task after a week of attempting SNS abstinence when compared to baseline. While this effect was opposite to the hypothesised direction, the strength of the correlation was weak and did not survive a Bonferroni correction for multiple tests (adjusted α = .017). No other significant correlations were found. Descriptive statistics for the EEfRT are displayed in Table 3.

**Table 3:** Descriptive stats for number of hard task choices (willingness to exert effort) and total number of keypresses (actual effort expenditure) in the EEfRT.

Measure	Minimum	Maximum	Mean	SD
*Pre-intervention*				
Hard task choices	0	12	7.43	3.38
Total keypresses	431	1108	766.76	165.08
*Post-intervention*				
Hard task choices	0	12	6.88	4.04
Total keypresses	398	1067	731.31	166.72

### SNS vs. control stimuli properties

Familiarity, valence and arousal rating for the SNS and control icons used in the VAAST and cue reactivity task were obtained at the end of the second lab session. Paired t-tests revealed that familiarity ratings for SNS and control stimuli did not significantly differ [*t(*50) = -0.40, *p* = .689, d = 0.06]. However, SNS stimuli were rated significantly higher on both valence [*t(*50) = 2.21, *p* = .032, d = 0.31] and arousal [*t(*50) = 7.55, *p* < .001, d = 1.10] measures. Therefore, while participants recognised the SNS and control app icons to the same extent, they reported liking the SNS icons more than controls as well as finding them more exciting.

### Compensatory behaviours

**Fig 4:**
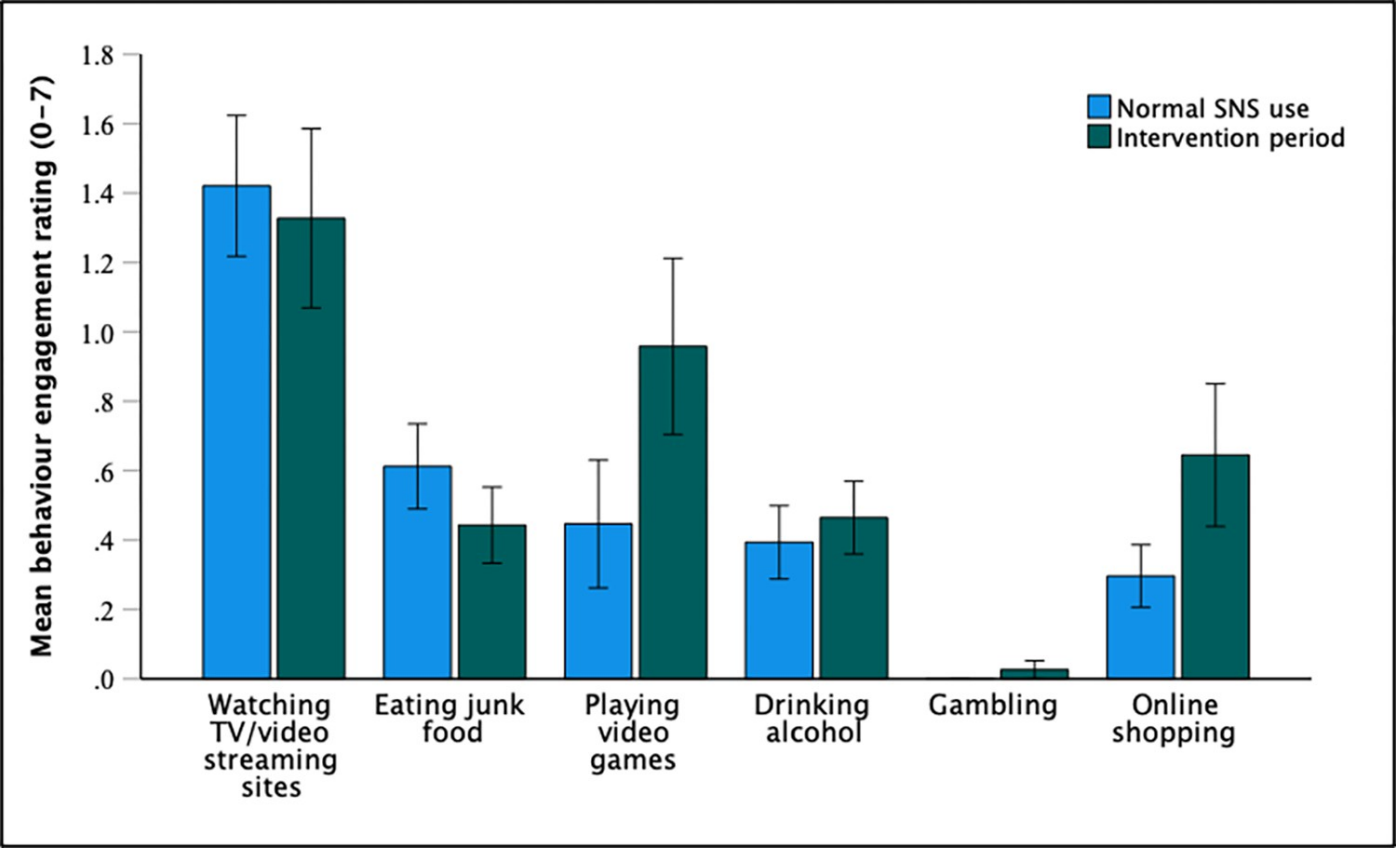
Mean Likert-scale responses to items assessing engagement with compensatory behaviours during normal SNS use vs. SNS abstinence. Error bars represent +/- 1 SE mean.

Compensatory behaviours were assessed in exploratory analyses by comparing self-reported engagement with other potentially problematic behaviours during the intervention phase versus periods of normal SNS use (i.e., baseline phase + post-intervention phase). Only responses from participants who responded to at least 50% of the end-of-day surveys during both the intervention phase and normal use phase were analysed. Consequently, the responses of 38 participants were included in the analyses. Paired t-tests revealed that participants reported spending significantly more time than usual playing video games during the intervention phase compared to periods of normal SNS use [*t*(37) = -2.08, *p* = .045, d = 0.34]. Participants also reported spending more time engaged with online shopping during the intervention phase, although this difference did not reach significance [*t*(37) = -1.72, *p* = .094, d = 0.28]. No significant difference in watching TV/video streaming sites [*t*(37) = 0.41, *p* = .682, d = 0.07], eating junk food [*t*(37) = 1.48, *p* = .147, d = 0.24], drinking alcohol [*t*(37) = -0.64, *p* = .529, d = 0.10], or gambling behaviours [*t*(37) = -1.00, *p* = .324, d = 0.16], were identified between periods of normal SNS use and SNS abstinence. Mean behaviour engagement responses are displayed in Fig 4.

## Discussion

The present study sought to assess potential changes in affect and motivation (both explicit and implicit) when restricting or abstaining from SNS use for one week. Ecological momentary assessments of mood were administered across a 15-day period, including baseline and post-intervention assessments which also allowed for the assessment of compensatory behaviours and rebound effects. Contrary to our pre-registered hypotheses but consistent with a growing body of recent research, we found no evidence that temporarily restricting SNS use produces withdrawal-like effects in SNS users or that such effects are correlated with problematic use symptoms. There was also no evidence to suggest that limiting SNS use is associated with clear positive effects on well-being. Instead of a generally negative or positive impact on mood, we observed partially opposing effects, with a reduction of positive affect from the baseline to the post-intervention period and a concurrent reduction of negative affect and boredom during the intervention. Importantly, reduced SNS use did not increase or decrease implicit motivation for SNSs (as indexed by approach biases, time distortion, and effort expenditure measured in two lab sessions pre- and post-intervention).

Analyses of screen time data showed that despite being willing to abstain from SNSs for one week, the vast majority of participants struggled to maintain abstinence, with 86.5% ‘relapsing’ at least once. High ‘relapse’ rates have also been observed in similar studies employing a SNS abstinence intervention [e.g., 18]. Such difficulties in maintaining abstinence despite initial willingness could be argued to be indicative of the addictive properties of SNSs. Yet on the other hand, almost all participants were able to significantly reduce their SNS use during the intervention period (mean use reduction of more than 80%) suggesting that users maintain some degree of control over their SNS behaviours. Furthermore, this reduction in SNS use had no consistent aversive effects on emotional well-being, which is contrary to expected effects during the experience of withdrawal. Given that SNSs are now so ingrained into normal everyday life and are often used to carry out essential communications it might not be helpful to interpret failures to comply with the abstinence instruction as ‘relapses’, especially since most participants were able to substantially cut down their SNS use. Additionally, unlike addictive substances, restricting SNS use had no effect on subjective cravings or measures of implicit wanting. This is also surprising as an increase in cravings is the most frequently reported abstinence-induced effect across all potential behavioural addictions [39]. Thus, our findings, along with most previous research [e.g., 30, 31–33, 37], suggest that most SNS users (even those with more excessive use or more problematic use) do not tend to experience psychological withdrawal-like effects when voluntarily limiting their SNS use for one week.

Interestingly, analyses of mood data indicated potentially offsetting effects of use reduction on mood, with concurrent decreases of both positive and negative affect. One potential account of the observed decrease of positive affect is a reduction of opportunities to seek and obtain social rewards on SNSs, including social approval through likes, positive comments, followers, etc. This is consistent with our recent work in which we have theorised that social reward is a key factor underpinning SNS use behaviours [58]. On the other hand, our data also indicated slight decreases in self-reported negative affect, with a specific dip during the intervention period. We speculate that such changes arise as a result of reduced exposure to subjectively negative experiences on SNSs, including processes related to the theoretical concepts of upward social comparisons [59] and Fear of Missing Out [60], or even bullying and harassment [61]. Future research should determine whether such concurrent and psychologically counter-acting reductions of both positive and negative social experiences are indeed characteristic of SNS abstinence and can potentially explain the lack of consistent effects on well-being in some recent studies [33–35]. Such a nuanced perspective on the emotional effects of limiting SNS use would also be consistent with the Goldilocks hypothesis of digital screen use, which posits that a moderate amount of SNS use may be beneficial to mental well-being [62]. However, we acknowledge that the analysis of the mood data in the present study was limited by removing participants who did not complete a sufficient number of EMAs and the lack of a control group–both aspects should be addressed in future work.

When examining usage behaviour across the three experiment phases both SNS and total screen time remained lower than baseline levels during the post-intervention phase, suggesting the absence of a rebound effect. It may be that participants developed strategies for limiting their SNS use during the intervention phase and that participants continued to implement some of these strategies when normal use was resumed. Therefore, temporary periods of restricted SNS use might be beneficial in helping to reduce use in the long-term. Consistent with this, one large-scale study found that participants who abstained from Facebook for four weeks were more likely to report using Facebook less or having quit altogether several weeks after the intervention [24]. However, there is also evidence that a rebound effect might occur in individuals who are more prone to experiencing greater disconnection during SNS abstinence [20]. Furthermore, our study also provides some evidence that individuals may engage more with other activities to compensate for their lack of SNS use. Participants reported spending significantly more time playing video games as well as a descriptive increase in online shopping during the intervention period compared to periods of normal SNS use. In another study where participants had to limit their SNS use to 10 minutes per day, they were found to spend more time using instant messenger apps and did not reduce their overall digital screen time [34]. However, while the present study allowed for the assessment of potential compensatory behaviours and rebound effects, these were only measured for the four days following the intervention period. Thus, we are unable to draw conclusion regarding the long-term effects that restricting SNS use may have on affect, motivation and screen time.

Our results using the AAT as a proxy of implicit SNS use motivation replicated those reported in our previous study [49], showing a large approach bias for SNS icons, relative to control stimuli. The present study demonstrated that this bias is unaffected by a week of SNS abstinence/use reduction, pointing to the robust nature of learned approach responses towards SNS stimuli. Consistent with our previous findings, we also observed an avoidance bias for control app icons, potentially indicating that these icons are devalued among regular SNS users when confronted with motivationally salient SNS stimuli. In the present experiment we also included post-experiment ratings of familiarity, valence and arousal. SNS and control app icons were rated as equally familiar by participants, ruling out an important confound of the present findings. In contrast to familiarity, SNS stimuli were rated significantly higher on both positive valence and arousal, confirming the high reward value of SNS stimuli and thus their capacity to elicit approach behaviour. Importantly, AAT scores did not differ between the pre- and post-intervention conditions. We acknowledge that the null effects of the intervention on motivation will need to be replicated in the future with a larger sample size. Given the importance of implicit mechanisms in addiction and the current lack of research investigating the relevance of these processes for problematic SNS use [45], future research should aim to incorporate more implicit measures to better understand the impact of SNS abstinence. At present, the interpretation of the absence of motivational changes is also hindered by the substantial amount of noncompliance with the abstinence instruction. Nonetheless, as previously noted, the considerable reduction in SNS use exhibited by the majority of participants can still be expected to produce effects on mood and motivation when such behaviour is considered to be problematic. However, it may be the case that participants were using SNSs ‘just enough’ to quell the negative consequences of non-use, providing a potential explanation for our null effects.

Interestingly, we did not observe the predicted variation of affective/motivational responses contingent on individual differences, including problematic use behaviours. However, conclusions regarding the lack of correlations between the assessed variables and problematic SNS use across our measures are weakened by the fact that we did not recruit from a ‘clinical’ population. It is possible that only individuals with extreme scores on assessments of problematic use exhibit withdrawal-like effects during SNS abstinence and thus more research sampling from clinical/treatment-seeking populations is required.

In sum, the present study indicates that abstaining or reducing SNS use for one week is not associated with any substantial effects on affective or motivational responses. Importantly, and contrary to our hypotheses, we found no evidence of withdrawal-like effects being correlated with more problematic SNS use. Our findings suggest that similar to recent consensus regarding the diagnostic guidelines for gaming disorder in the ICD-11 which have eschewed withdrawal criteria [63], the concept of withdrawal may also be less important for the diagnosis of problematic SNS use. However, our results also provide evidence that SNS use reduction has subtle and potentially offsetting effects on mood and that urges to use SNSs are a robust element of motivational hierarchies in individuals exposed to modern technologies.
